# Racialized migrant women’s discrimination in maternal care: a scoping review

**DOI:** 10.1186/s12939-025-02384-8

**Published:** 2025-01-20

**Authors:** Jasmine Therese Arcilla, Alexandra Nanou, Sarah Hamed, Fatumo Osman

**Affiliations:** 1 School of Health and Welfare, Dalarna University, Falun, 79 182 Sweden; 2 Sustainability Learning and Research Center (SWEDESD), Department of Women’s and Children’s Health, Uppsala University, Akademiska Sjukhuset, Uppsala, 75 185 Sweden; 3 Centre for Gender Research, Uppsala University, Box 527, Uppsala, 75 120 Sweden; 4 King’s College London Institute of Psychiatry, Psychology & Neuroscience (IoPPN) IoPPN, 16 De Crespigny Park, London, SE5 8AB England; 5 Black Thrive Global CIC 167 – 169 Great Portland Street, London, W1W 5PF England

**Keywords:** Migrant women, Racialized, Discrimination, Maternal healthcare, Barriers, Intersectionality, Racism

## Abstract

**Background:**

Despite equality and quality being the core of good healthcare, racial and ethnic inequalities continue to persist. Racialized groups, including racialized migrant women, experience various forms of discrimination—particularly during maternal care encounters, where intersectional forms of discrimination may occur. Experiences of discrimination in maternal care have been associated with poor health-seeking behavior and adverse maternal health outcomes. However, research on racialized migrant women’s discrimination in maternal care is limited. This scoping review aims to give an overview of the state of current research on the discriminatory experiences of racialized migrant women when utilizing maternal healthcare and its gaps to ensure equity in global maternal healthcare.

**Methodology:**

This scoping review mapped out all available English-language scientific empirical literature published between 2012 and 2023. All authors agreed on the inclusion criteria. Collecting, charting, and reviewing the included material were done using the 2018 Preferred Reporting Items for reviews and Meta-Analyses extension for Scoping Reviews (PRISMA-ScR) checklist. The search strategy included electronic databases, such as Pubmed, CINAHL, MEDLINE, Web of Science, and PsycInfo.

**Results:**

A total of 57 articles were included and analyzed. The majority were qualitative and conducted in European and North American countries. None of the included article’s aims originally intended to focus on discrimination. However, their findings exposed the many ways racialized migrant women experienced discrimination when using maternal healthcare services—from accessibility problems, non-utilization of interpreters, and untimely and delayed care to disrespect, abuse, and differential care. Racialized migrant women’s discrimination resulted in a lack of agency and being excluded from decision-making.

**Conclusions:**

While the included articles allude to some issues related to discrimination in maternal healthcare experienced by racialized migrant women, this review delineated knowledge gaps warranting discussion. Few articles focus on and conceptualize discrimination from a racialized lens in maternal healthcare. A limited geographical scope in research and knowledge generation on discrimination and racialization exist in this field as does a lack of sufficient articles on discrimination and racism from healthcare personnel. Lastly, many of the existing studies lack an intersectional lens in exploring discrimination in maternal care against racialized migrant women.

**Supplementary Information:**

The online version contains supplementary material available at 10.1186/s12939-025-02384-8.

## Background

Notwithstanding that equality is a core ethic of healthcare, racial and ethnic inequalities continue to persist. These inequalities are detrimental to the health of racialized minoritized people (referred to henceforth as racialized people for simplicity reasons while maintaining the minoritizing nature of racialization) resulting in adverse health outcomes, which include increased mortality rates, such as the case of maternal mortality among racialized migrant women across various European countries [1]. We define racialized people in this review as groups of people who are racialized as inferior in contrast to the dominant group and consequently minoritized and hence subjected to unequal power-relations in different institutions. The term racialized is used to refer to a dynamic ongoing process of racialization which we define as a sociohistorical process rooted in colonial domination, whereby groups of people are stratified somatically and culturally within groups of subordination and supraordination [2]. Racialization results in discrimination across and within various institutions in nation-states including healthcare. In this review, discrimination is defined as a socially structured phenomenon justified by an underlying process (of racialization) manifesting in interactions among individuals and between individuals and institutions, maintaining the advantages of the dominant group at the expense of the minoritized group [3]. Discrimination in healthcare contributes to suboptimal care, differential diagnosis and treatment, as well as loss of trust in healthcare, thus, exacerbating the burden of poor health [4]. Due to these vast ethnic and racial inequalities, many leading public health outlets have highlighted these inequalities as a public health crisis [5]. In this study, we use the term migrant to refer to an umbrella term reflecting the common understanding of a person who moves away from their usual residence, whether within a country or across an international border, temporarily or permanently, and for different reasons. The term includes several well-defined legal categories of people, such as migrant workers; persons whose particular types of movements are legally defined, such as smuggled migrants; as well as those whose status or means of movement are not specifically defined under international law, such as international students [6].

While research documents how discrimination contributes to suboptimal care among various racialized groups, research on migrants’ experiences of discrimination is underwhelming in comparison to other causes of inequalities, such as communication and language [7]. Even though research on discrimination against racialized migrants in healthcare is fragmented, current studies allude to the existence of implicit and explicit anti-migrant attitudes among healthcare personnel with othering documented as a common form of anti-migrant bias, particularly prominent against racialized migrants. For instance, a recent quantitative study from Belgium found that most of the studied general practitioners exhibited implicit bias against ethnic and racialized migrant groups [8]. Racialized migrants also discuss being scrutinized and not taken seriously by healthcare personnel, who devalue their identity [7]. For example, a study in Norway has shown that sub-Saharan migrants experience differential treatment by healthcare personnel, such as being treated as second-class citizens and being dismissed from decision-making processes [9]. Moreover, studies also show that racialized migrants are subjected to prejudicial assumptions that portray them as difficult and frustrating and their symptoms as unworthy of care, leading to differential medical outcomes [4, 7].

Among the various racialized migrant groups, women are particularly vulnerable given the intersectional nature of their experiences—women of a racialized minoritized group—living in a foreign country. This vulnerability is exacerbated in the context of maternal care due to the various forms of discrimination that exist. Existing research documents how discrimination and the over medicalization of maternal health services through historical Eurocentric medical practices is reflected in the experiences of women accessing maternal care. According to a recent review [10], obstetric violence is prevalent across national settings globally. For instance, a study in Mexico showed that in 2015, 23.6% of women aged 15–49 years old who had given birth in the previous five years had experienced some form of obstetric violence [11]. Other studies, for example from the USA, demonstrate similar results. In 2019, a US study showed that 17.3% of women in a national survey who were pregnant between 2010 and 2016 experienced some form of obstetric violence including physical abuse, sharing of private information without consent, having their physical privacy violated and other forms of disrespect [12]. Obstetric violence has also been reported in various contexts across the Global South including in different African countries such as Kenya [13] and Tanzania [14], where women reported physical abuse, non-consensual care and detainment for non-payment of fees. A systematic review from India [15], revealed that obstetric violence is mostly prevalent in the form of verbal abuse followed by physical abuse and other forms of abuse. The review also showed that this violence is exacerbated by other forms of oppression including class, being from a religious minoritized group and socioeconomic status (ibid).

Obstetric violence has been discussed extensively in various contexts across Latin America resulting in political movements for the humanization of childbirth and the inclusion of obstetric violence as a legal term in countries such as Venezuela [16]. Recent research has also shown that the historical racialization aspect of obstetric violence aggravates the violence that racialized women encounter in healthcare, a process that has been dubbed as obstetric racism which lies at the intersection of racialization and obstetric violence [17]. Research combining different intersecting categories and realities (i.e., being a woman and of a racialized migrant background accessing maternal care) of discrimination is underwhelming especially in comparison to other causes of inequalities like communication and language [7]. Studies from Europe show that racialized migrant women, particularly refugees and undocumented migrants, have higher risks for poor maternal self-rated health and adverse infant outcomes [18]. Moreover, research also reports significantly higher risks for maternal and perinatal mortality among racialized migrant women compared to native majoritized women [19]. Further, studies also show how healthcare personnel’s use of racialized discourse stigmatizes racialized migrant women, especially sub-Saharan African women, as shown by a Norwegian study [20]. In another study, on maternity care in Ireland, providers used an “us” and “them” racialized discourse to homogenize racialized migrant women of Asian descent [21]. A recent scoping review in the UK on discrimination against racialized migrant women in maternity care showed that women experienced healthcare personnel as rude, discriminatory, and insensitive to their cultural and social needs [22]. These experiences of discrimination in healthcare have been shown to affect how racialized women use maternal and perinatal care, such that they often avoid or delay seeking care even when needed [23].

Since negative social determinants—such as material deprivation, extensive restrictions on social security, welfare, and healthcare, as well as subjection to discrimination by citizens, local authorities, and social service providers—threaten racialized migrant women’s maternal health [24], it is paramount to understand the state of current research and its gaps to ensure equity in global maternal healthcare. In this work, we consider how experiences of discrimination manifest and reflect on how racialized women access and use maternal healthcare. We also consider situations wherein racialized migrant women perceive that they receive suboptimal maternal healthcare—in comparison to their local counterparts—as discrimination as well. Thus, this study’s overall aim is to conduct a scoping review examining the discriminatory experiences of racialized migrant women when accessing maternal healthcare by gathering evidence from the existing empirical literature.

## Methodology: design and method

### Design

This scoping review includes published scientific literature deemed relevant to the research aim. The collated literature is mapped out to provide an overview of the volume and depth of the available scientific resources on the topic and to identify gaps that need to be filled or clarified [25]. The Preferred Reporting Items for Systematic reviews and Meta-Analyses extension for Scoping Reviews (PRISMA-ScR) checklist guided this scoping review [26].

### Eligibility criteria

To ensure the relevance of the studies, literature found between 2012 and 2023 were included. In connection to this timeframe, we observed during the literature search that published articles in this area significantly increased only after 2012. On top of the aforementioned pragmatic reasoning for the timeframe, Hamed et al. [27] scoping review on racism in healthcare presents a graph depicting the sharp increase after 2012 in published articles on racism within the global health sector–reflecting the point in time wherein there was a drastic increase in academic interest on this issue [27]. Moreover, we also chose 2012 as a starting point due to the drastic increase in the number of migrants from the Global South to the North due to the so-called migration crisis which shifted the discourse on migration in Global North countries. This year was, thus, deemed an even more relevant basis for the start of this study’s inclusion interval. Additionally, only English-language studies were reviewed because of practical reasons such as the time and resources needed to translate other languages as well as the authors’ language proficiency limitations.

The target population for literature inclusion is racialized migrant women. For this scoping review, we use the general term “migrant” while understanding it covers categories, including refugees, asylum seekers, and undocumented migrants. We depart from the IOM’s [6] definitions of these various groups of migrants. Thus, for this article, a refugee is “*a migrant who owing to a well-founded fear of persecution for reasons of race, religion, nationality, membership of a particular social group or political opinion, is outside the country of his nationality and is unable or, owing to such fear, is unwilling to avail himself of the protection of that country; or who, not having a nationality and being outside the country of his former habitual residence as a result of such events, is unable or, owing to such fear, is unwilling to return to it*” [6]. An asylum seeker is defined as “*an individual who is seeking international protection. In countries with individualized procedures, an asylum seeker is someone whose claim has not yet been finally decided on by the country in which he or she has submitted it. Not every asylum seeker will ultimately be recognized as a refugee, but every recognized refugee is initially an asylum seeker*” [6]. Given the large portion of migratory movement observed is from a country with fewer opportunities to one with greater chances of having a better quality of life, this study also focused more on inter-country or international migration, rather than intra-country or domestic migration. Since the research is studying the discriminatory experiences of migrant women while seeking and utilizing regular maternal health services in their host country, internally displaced people, or women residing in refugee camps were excluded. This is because of the added special (camp) situation, which has too much instability. Hence, refugee camps do not reflect the natural situation of maternal healthcare services in the host country. Literature focusing on pregnant migrant teenagers was also removed, as their age added another compounding variable of discrimination outside the regular delivery and utilization of migrant maternal healthcare. Other criteria, such as specific countries of origin or host countries of migrant women, were not imposed in the review. These criteria were omitted to ensure more variety and diversity in the included literature. Perspectives of healthcare service providers, family members, and other external persons were also included as long as they were taken alongside the migrant women’s experiences. Lastly, studies using a review-type methodology (scoping, systematic, etc.) and dissertations were unanimously decided to be removed in the latter portion of the filtering process to reduce redundancy in the synthesis and to ensure the works included were peer-reviewed and quality-checked. All these criteria are summarized in Table 1.

**Table 1:** Inclusion and exclusion criteria

Inclusion Criteria	Exclusion Criteria
Experiences/perspectives of racialized migrant women (including articles with family members and healthcare personnel)	Teenage migrants
Experiences in health facility settings and while receiving maternal health care	Unstable living conditions (e.g. refugee camps, detention centers)
Empirical studies	Review methodology
Peer reviewed work	Dissertations or non-peer reviewed work
Studies from 2012–2023	Studies only providing healthcare personnel ‘s perspective
English language	Internal migration/displacement
All income levels of countries	

### Search strategy

The literature search began in September 2023 to identify relevant studies through searching electronic databases and sources. PubMed, CINAHL, MEDLINE, Web of Science, and PsycInfo were used. The following combination of search words was uniformly used for all databases according to the Joanna Briggs Institute (JBI) Manual for Evidence Synthesis [28] general format of population, concept, and context (PCC). The population was searched in the “abstract” field as “*refugee or migrant or immigrant or asylum seeker*” combined with the concept of “*maternal health or pregnancy or perinatal health*” using the boolean operator “AND.” The context was searched in the “all text” field as “*discrimination or racism or bias or prejudice or stigma or stereotype*,” with additional terms of “*experiences or perceptions or views or attitudes*” using the boolean operator “OR.” The results of the population and concept search were then combined with the results of context one through the “AND” operator.

#### Article selection

The articles collected in the first search were narrowed down by filtering them using their titles, their abstracts, and the content of their entire texts. The aim, inclusion, and exclusion criteria constituted the basis for deciding which literature to include and remove from the list. An initial list of 1,013 article titles, created through an online spreadsheet (Google Sheets), was shared among all four authors and split evenly for each author to have between 253 to 254 titles to assess which would have their abstracts read. To ensure the quality of the review process, we consistently checked one another’s work; one author was assigned to review the articles that the other author had been assigned to read regarding checking the titles chosen to proceed to the abstract reading. Concerning the more ambiguous titles, we discussed them during joint online discussions to establish whether they would be included or not. A process was conducted for the succeeding stages when the group filtered the abstracts (374 articles) and entire texts (72 articles). The open-source reference management software Zotero was used for easier sharing of the full-text versions of the articles.

A second strategy was originally planned using the reference lists or bibliographies of the articles obtained from the database search. However, upon reviewing these lists, we found that the relevant studies were already included and filtered through during the first search strategy process. Thus, we agreed that a saturation point had been reached, since no new relevant literature had arisen due to the large number of literatures already included.

#### Data extraction

Data were charted from November 2023 to the end of March 2024 by sorting the collected material according to the general profiles of each article. The details included in the charting process were inspired by Arksey and O’Malley’s [29] original scoping review study. Information such as the name of the author(s), intervention type, study population, study context, methodology, and key findings were all organized, tabulated, and tallied using the aforementioned online spreadsheet. Additional categories were included in the table to help layout and better visualize each study’s characteristics. These are the study aim, methodology, host country in which the studies were done, migration term used, and the specific service provider discriminating against migrant women (see Supplementary Table 1).

#### Data analysis

Using the thematic analysis method by Braun and Clarke [30], we used the key findings of the included articles on discrimination to develop codes to build the types of discrimination experienced by migrant women. The coding process was led by researchers J.A and A.N., who evenly split the 72 articles to develop the codes for each. The codes were based on the key findings tabulated in the spreadsheet developed by the group. Both researchers cross-checked each other’s work to ensure consistency in the generated codes. The common codes found among the articles were later segregated into larger, more study-specific themes, presented in the following results section. These codes and subthemes were then presented to the rest of the researchers, F.O. and S.H., to ascertain each’s quality, relevance, and distinctiveness. The codes and themes needing refinement were revised accordingly.

## Results

### Included studies

The initial number of articles gathered from the five databases was 1,995. After removing duplicates with the help of the Zotero software, this number was reduced to 1,013. These articles were first filtered by their titles according to the exclusion and inclusion criteria, which reduced the number to 374. The same process was done with the abstracts of the remaining items, which resulted in 72 articles needing to be retrieved and read through completely.

After retrieving and reading the texts, we excluded an additional 15 studies. One was excluded because the entire article was not retrievable. Two were removed due to being conducted in unstable contexts, such as refugee camps, detention centers, dispersals, or internal displacement situations. Another five were excluded because their focus was not on the discriminatory experiences of migrant women while accessing maternal healthcare services. Six studies were removed because of their research formats or methods: one conference abstract, two dissertations, and four scoping or systematic review studies.

**Fig. 1:**
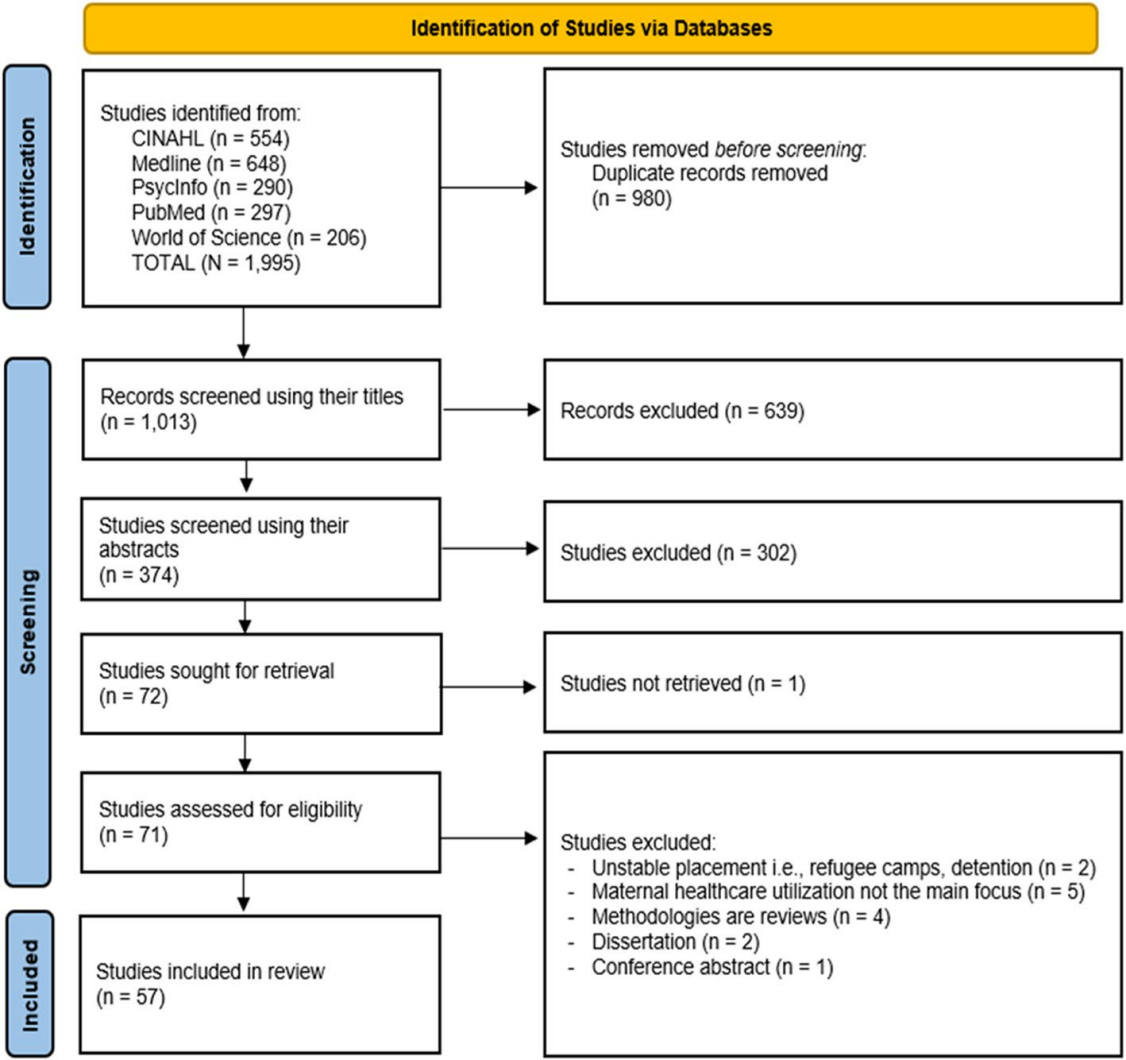
Search strategy process of the study

In total, our search yielded 57 articles to include in this review. Figure 1 summarizes this search process.

The results section is divided into two main parts. The first part includes the characteristics of included studies consisting of the study designs, study aims, discriminating healthcare personnel, migrant groups mentioned in the included studies, and host countries. The second part presents the thematic findings deduced from the analysis of the included studies using Braun and Clarke’s [30] thematic analysis. The analysis resulted in three main themes: systemic Issues, interactions with healthcare personnel and quality of care, and consequences of discriminatory experiences.

### Characteristics of included studies

#### Study designs

Qualitative, quantitative, and mixed empirical studies were included, with qualitative studies being the most common. Overall, we included two mixed-methods studies [31, 32], one multi-case study [33], ten quantitative studies [34–43] and 44 qualitative studies [44–87].

#### Study Aims

Studies were included if they reported on discrimination. However, none of the included studies’ original aims contained the word “discrimination” and hence did not set out to primarily investigate discrimination as their main aim, notwithstanding their reporting on discrimination. The aims in 8 out of the 57 articles included comparing racialized migrants and their local counterparts [34, 37, 39, 41–43, 45, 75]. One study conducted in the Netherlands [75] and one in Portugal [45] applied qualitative methodology while the rest were quantitative studies. The quantitative studies were conducted in Norway [34, 41], the United Kingdom (UK) [39], Finland [42], and Australia [43]. One study [37] included 11 countries in the European region: Sweden, Italy, Norway, Slovenia, Portugal, France, Germany, Croatia, Serbia, Switzerland, and Luxembourg. One study [52] compared racialized migrant women’s experiences of maternal care in two different countries: Norway and the United States (US).

In total, 22 studies concerned racialized migrant women’s experiences of maternal healthcare services in the host country. All studies applied qualitative methodology and were conducted in Portugal [48], Sweden [49, 69], Switzerland [50, 78], Denmark [57], Canada [61], Norway [63, 71, 83], the UK [67, 68, 73], the US [70, 74, 85], Iran [72], Australia [77, 87], Austria [81], and Türkiye [84, 86].

#### Discriminating Healthcare Personnel

The healthcare personnel occupational category mostly mentioned by racialized migrant women in the included articles were physicians (*n* = 28) [32, 34, 40, 43–45, 47, 48, 50, 51, 53, 56, 62, 64–68, 70, 72, 75–79, 82, 85, 86], midwives (*n* = 24)[32, 34, 35, 40, 43, 44, 48–50, 52–55, 60, 63, 66–69, 71, 72, 75, 79, 84] and then nurses (*n* = 6) [52, 77, 79, 81, 82, 85]. The least mentioned occupational category was administrative personnel (*n* = 3) [45, 80, 84].

#### Migrant groups mentioned in the included studies

The articles used various migrant group categories. Almost half of the articles (n = 27) used the term “migrants” to refer to their study population [31–40, 44–46, 48–51, 56, 60, 65, 67, 69, 72, 73, 78, 80, 83],while 21 articles used the term “immigrant” [41, 43, 47, 52, 53, 57, 58, 61, 63, 64, 66, 68, 70, 71, 75, 76, 79, 81, 84, 85]. Eight articles focused mainly on “refugees” [53–55, 62, 77, 82, 86, 87] and one on “asylum seekers” [59]. Six studies added the specific characteristic of being “undocumented or illegal” in conjunction with the terms “migrant” or “immigrant” [42, 45, 49, 51, 57, 73].

#### Host Countries

**Fig. 2:**
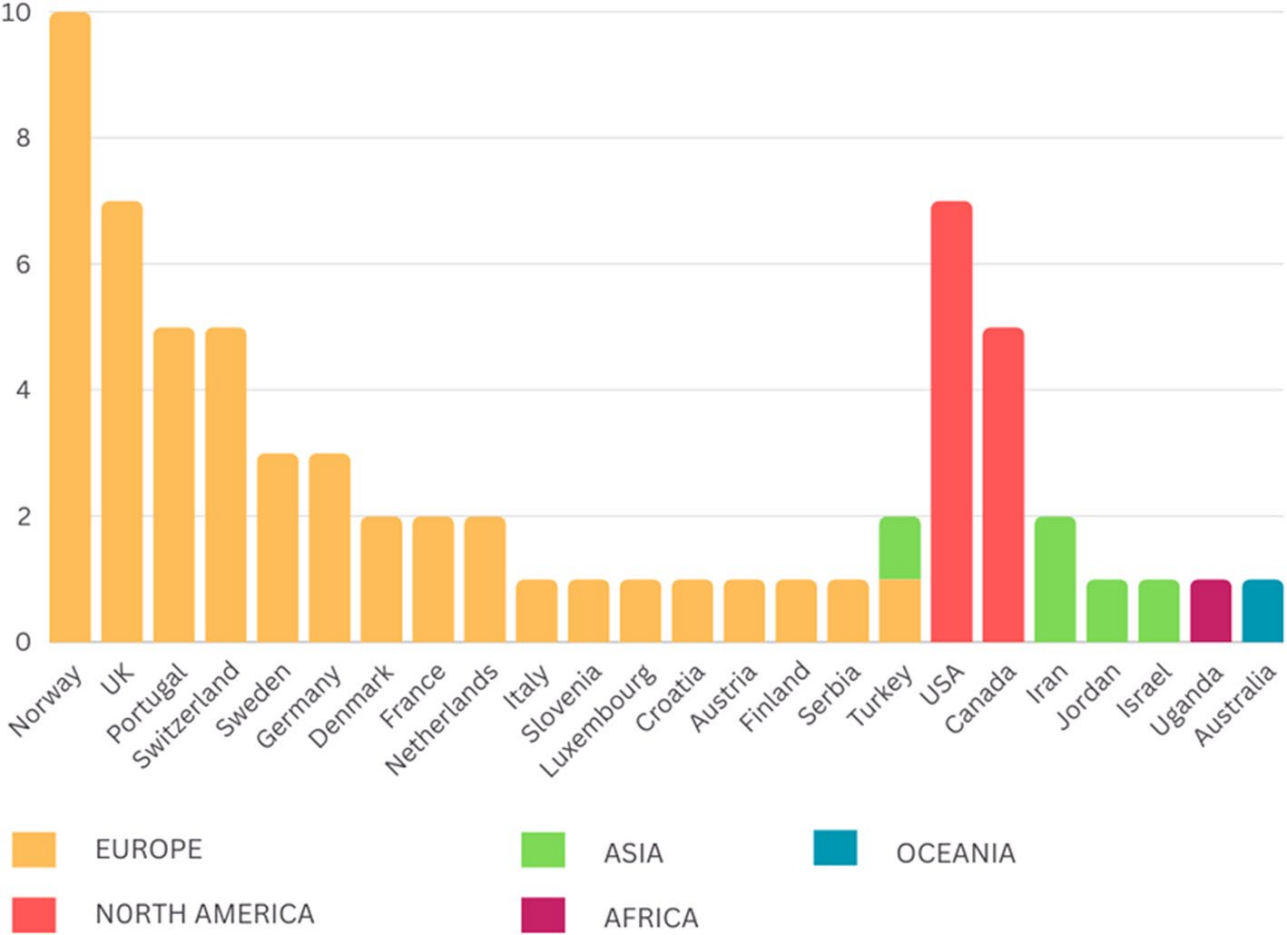
Countries where the studies were conducted

The host countries were categorized based on their geographical regions (see Fig. 2). Sixteen countries were in Europe: Norway [31, 34–37, 41, 63, 71, 83], the UK [33, 39, 55, 60, 67, 68, 73], Portugal [37, 38, 45, 46, 48], Switzerland [37, 50, 65, 78, 80], Sweden [37, 49, 69], Germany [37, 56, 62], Denmark [57, 66], France [37, 79], Netherlands [32, 75], Italy [37], Slovenia [37], Luxembourg [37], Austria [81], Finland [42], and Serbia [37]. Two studies were conducted in Türkiye [84, 86], which is a transcontinental country spanning Asia and Europe. Three countries were in Asia: Iran [53, 72], Jordan [82], and Israel [59]. In North America, studies were conducted in the US [44, 51, 52, 63, 70, 74, 85]and Canada [40, 58, 61, 64, 76]. Only one study was conducted in Africa: Uganda [47]. Lastly, four studies were conducted in Oceania: Australia [43, 54, 77, 87].

### Thematic findings

**Fig. 3:**
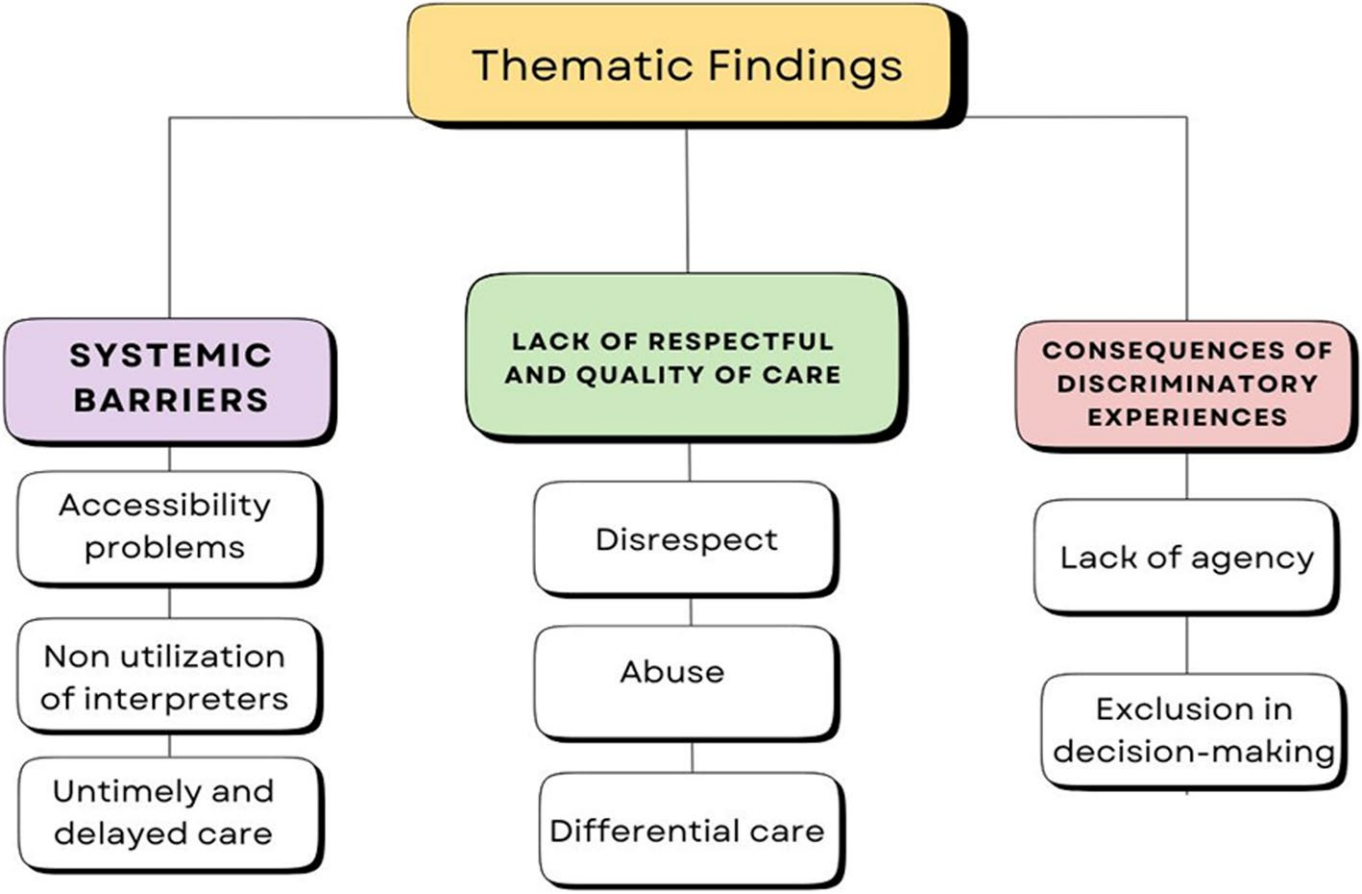
Thematic findings with the main themes and sub-themes

Different forms and consequences of discrimination were reported in the articles. In general, the studies’ results related to discrimination fell under the following main themes: systemic barriers, lack of respect and quality of care, and consequences of discriminatory experiences. These main themes were identified to capture the three possible stages that racialized migrant women go through while accessing maternal healthcare wherein they face and perceive discrimination. These were further classified under sub-themes, which are illustrated in Figs. 2 and 3. Under systemic barriers, accessibility problems, non-utilization of interpreters, and untimely and delayed care were identified. Concerning lack of respect and quality of care, disrespect, abuse, and differential care were also identified. Lastly, for consequences of discriminatory experiences, lack of agency and exclusion from decision-making were identified.

#### Systemic barriers

The forms of discrimination under this theme relate to racialized migrant women’s experiences of discrimination before or at the point of accessing maternal healthcare services and not only during the actual utilization of care.

#### Accessibility problems

Amongst the three barriers, those limiting accessibility to services were the most common, with 30 studies [35–37, 42, 45, 46, 49, 51–54, 56–59, 66–70, 72–74, 76, 78, 80, 82–84, 86] reflecting this issue. The host countries involved in these studies were Norway [31, 36, 37, 52, 83], Portugal [37, 45, 46], the US [51, 52, 70, 85], the UK [67, 68, 73], Switzerland [37, 78, 80], Sweden [37, 49, 69], Iran [53, 72], Denmark [57, 66], Canada [58, 76], Germany [37, 56], Türkiye [84, 86], Israel [59], Finland [42], Jordan [82], Italy [88], Slovenia, [88], Luxembourg [88], France [88], Croatia [88] and Serbia [88]. Out of the 30 articles that were identified to have access issues, 26 of them were qualitative. Those that had different methodological designs used either quantitative [36, 42, 88] or mixed methods [31].

Racialized migrant women in 15 out of the 57 included studies mentioned that they needed to negotiate their right to maternal healthcare services, either directly with personnel or by fulfilling requirements first—such as having healthcare insurance, legal residency status or being a married woman—to get the same treatment as their local counterparts (15/57) [31, 36, 42, 45, 49, 51, 57, 73, 74, 76, 78, 80, 83, 84, 86]. One of the findings in a 2020 study by Funge et al. [57] in Denmark concerning the experiences of undocumented migrant women originating from the Philippines, Sudan, Morocco, Pakistan, Kenya, Tanzania, Uganda, and Bosnia, with access to maternity care services, specifically highlights this issue:

“*When the women needed care in the early stages of labor, or in relation to the induction of labor, some experienced that they had to negotiate their entitlement to care with health professionals, making the women feel neither welcomed nor acknowledged … For post-natal care, the women experienced being questioned about entitlement to care and about their living conditions.”* ([57], p5).

Another access barrier frequently mentioned is the interference of matters external to healthcare, such as lack of legal (migration) documents and permits, as well as the social and cultural responsibilities and expectations like prioritizing household work and their families. These matters make it difficult for racialized migrant women to begin or continue using maternal healthcare services (10/57) [31, 46, 53, 56, 58, 59, 66, 73, 74, 76]. A study conducted in Denmark [66] interviewed non-Western immigrant women and aimed to investigate the everyday life situations affecting the response to pregnancy complications. The article included the testimony of one woman and how she prioritized fulfilling her expected social responsibilities but ending up with health repercussions by doing so:

*“My spouse told me, you can’t go to work, you were ill yesterday. I tell him no! I have to go to work because two of my colleagues were off work ill … It won’t work if I stay home … so I went to work... when I came home it was really bad, a lot of pain in my stomach … I had pain for four to five minutes at a time. I have never tried that before, I got scared, will I deliver now, before my due date?”* ([66], p8).

In some studies, racialized migrant women shared that they experienced certain financial constraints that made the entire process of accessing and utilizing maternal healthcare more unaffordable for them (7/57) [53, 57–59, 72, 73, 80]. For instance, a study in Iran showed that the combination of more costly maternal healthcare services coupled with the lack of proper health insurance reported significant delays for Afghan migrant women living in Iran to seek appropriate care [72]. The most concrete form of barred access was seen in nine studies in which women experienced direct denial or exclusion from maternal healthcare—meaning they were not allowed to access it at all regardless of their situation or bargaining. These studies were conducted in Norway, Sweden, Iran, England, the USA, Canada, Finland, and Jordan (9/57) [31, 42, 49, 68, 72–74, 76, 82]. In Pimienta et al.’s recent 2023 study [76] on newcomer women’s experiences with perinatal care during the three-month health insurance waiting period in Ontario, Canada, the authors shared a story about one woman who was directly turned away from prenatal services multiple times because of her immigrant status.

In the instances that racialized migrant women were granted access to the maternal care they needed or sought, some have accounted that they were given access to suboptimal or inadequate services and systems (13/57) [33, 36, 45, 49, 52, 54, 59, 67–70, 72, 88]. In some studies, this was seen in comparison to their expectations and experiences from their home country [45, 68] or to the host country’s expected and accepted maternal healthcare standards [33, 37, 59, 72].

#### Non-utilization of interpreters

The issues of the language used during service provision and the availability of interpreters were also significant systemic barriers that frequently surfaced in the collated studies (23/57) [31, 36, 38, 39, 42, 45, 50, 52, 55, 59, 62, 65, 67, 71, 74, 76–78, 80, 83, 84, 86, 87]. The countries in which the studies were conducted were Norway [31, 36, 52, 71, 83], Switzerland [50, 65, 78, 80], the UK [39, 55, 67], Portugal [38, 45], the USA [52, 74], Australia [77, 87], Türkiye [84, 86], Israel [59], Germany [62], Canada [76], and Finland [42]. The majority of the identified studies in this sub-theme employed qualitative methodology (18/23) while four studies [36, 38, 39, 42] applied a quantitative approach and one study [31] applied a mixed-methods methodology.

In the articles included in our scoping review, these language issues occurred because of the host country not providing access to interpreters or translated material for racialized migrant women. In cases where interpreters were available, many racialized migrant women still mentioned that they were not offered interpreter services, or when they were, it was not standardized for all [38]. This resulted in some racialized migrant women finding their own interpreter in the form of family members, friends, or other patients in the clinic—who were all unlikely able to capture important medical terms and processes sufficiently [59]. Many were also excluded from prenatal courses because they were not offered in other languages that the women were more familiar or comfortable with [31, 50].

#### Untimely and delayed care

The last systemic barriers highlighted frequently in the included studies were time-related ones faced while accessing maternal health services (17/57) [31, 35, 38, 40, 42, 45, 46, 56, 58, 64, 66, 69, 72, 79, 86–88]. Specifically, untimely and delayed care was mentioned in studies conducted in Portugal [38, 45, 46, 88], Norway [31, 35, 88], Canada [40, 58, 64], Sweden [69, 88], Germany [56, 88], France [79, 88], Luxembourg [88], Switzerland [88], Slovenia [88], Croatia [88], Italy [88], Serbia [88], Denmark [66], Iran [72], Finland [42], Türkiye [86], and Australia [87]. Five out of these studies employed a quantitative methodology [35, 38, 40, 42, 88], and one study used a mixed-methods approach [31]. The rest of the related studies were qualitative.

Fifteen studies specifically mentioned that racialized migrant women did not receive timely treatment or had longer waiting times in comparison to their non-migrant counterparts (15/57) [31, 35, 38, 40, 42, 45, 46, 56, 58, 66, 69, 72, 79, 87, 88]. Those studies were geographically located in Portugal, Norway, Germany, Canada, Denmark, Sweden, Iran, France, Finland, and Australia. One particular study comparing the perinatal experiences of “African immigrants” to their French counterparts clearly showed the forms of delayed care that the former had to go through. This study by Sauvegrain et al. [79] (2017) highlighted how immigrant African women needed multiple repeats of urine testing for proteinuria, compared to French women, before getting a diagnosis of preeclampsia, thus also delaying the start of their treatment. Four studies also reported that racialized migrant women were rushed or had too short of a conversation with their healthcare providers during their treatment (4/57) [35, 46, 64, 86].

#### Lack of respect and quality of care

In this sub-theme, we grouped the discriminatory experiences of racialized migrant women during interactions with healthcare personnel while accessing maternal healthcare services. Three sub-themes were identified: disrespect, abuse, and differential care.

#### Disrespect

Racialized migrant women shared experiences of disrespect when interacting with healthcare personnel in the vast majority of the included studies (48/57) [31–33, 35, 36, 38–41, 43–46, 48–55, 58–61, 63–78, 80–86]. The geographical locations within which these studies were conducted were Norway [31, 35, 36, 41, 52, 63, 71, 83], the UK [33, 39, 55, 60, 67, 68, 73], the US [44, 51, 52, 70, 74, 85], Canada [40, 58, 61, 64, 76], Portugal [38, 45, 46, 48], Switzerland [50, 65, 78, 80], Australia [43, 54, 77], Sweden [49, 69], Iran [53, 72], the Netherlands [32, 75], Türkiye [84, 86], Israel [59], Denmark [66], Austria [81], and Jordan [82]. As far as the methodological design is concerned, the studies included in this sub-theme, for the most part, used qualitative designs (39/48). Two were mixed-method studies(31, 32), one was a multi-case study [33], and the rest were quantitative studies (6/48) [35, 36, 39–41, 43].

Fourteen studies [32, 39, 40, 46, 48, 49, 54, 69, 73, 74, 80–82, 84] included testimonies of racialized migrant women who were met by an unwelcoming attitude or disrespectful behavior from healthcare personnel. For instance, a racialized migrant woman seeking care in Sweden shared the outcome of being late to her appointment: *“We arrived a little late, she got extremely angry at us and told us off. She said ‘why did you come, we have other things to do’”* ([49], p8).

Apart from the uninviting environment, in 16 articles (16/57), healthcare personnel did not acknowledge racialized migrant women as capable of having control over their bodies and lives. In these studies, women disclosed that they were not taken seriously by healthcare personnel [40, 48–51, 54, 58, 59, 61, 66, 72, 75, 85]. One example was presented in Cerdeña’s [51] person-centered ethnography study in southern Connecticut in which a woman received a contraceptive implant insertion without giving her consent. The woman felt like she was having “a needle in her hand” and therefore visited the prenatal care safety net clinic to have it removed: “…*They told me that everything was fine, that nothing was wrong, and that they didn’t have to remove it*” ([51], p7)*.* On top of not being taken seriously, in four studies, healthcare personnel intervened outside the healthcare sphere [32, 52, 59, 69]. Racialized migrant women shared that the personnel would focus on their lifestyle rather than their health. For example, a racialized migrant woman in Sweden reported that her midwife was not interested in her pregnancy or her health; rather, the midwife focused on the number of children the couple had and made suggestions concerning sterilization, education, and work [69].

Not only did migrant women feel that the healthcare personnel found themselves superior to them when it came to knowing their bodies or handling their lives [32, 45, 60, 65, 67]. Moreover, according to the experiences of racialized migrant women, this led to the delivery of impersonal care [32, 43, 50, 63, 65, 68–71, 75, 76, 83]. For instance, a woman during a discussion around healthcare providers’ control over birth and birth outcomes in the Dutch context disclosed: *“How can I talk to the midwife and feel that we have an honest conversation, when she projects her truth about me but does not want to learn from me or about me?” *([32], p7).

In addition, it was noted that healthcare personnel had prejudices towards their patients in thirteen of the collated articles (11/57) [31, 32, 51, 53, 60, 67, 69, 72, 82, 84, 85]. A Somali migrant woman who used maternal care services in the UK context shared the following:

“*Maybe because of our color, culture, religion or some other reason, they [staff] think they are better than us. Sometimes you will hear women being abused by comments like, you are coming back every day because you want to live on the benefit money. The worst thing one staff said was ‘I will probably see you again next year’… it makes you feel worthless*.” ([67], p4).

In 24 studies (24/57), racialized migrant women reported being neglected by healthcare personnel or that they were not making an effort to show care [32, 36, 39–41, 45, 48, 49, 51, 52, 54, 55, 59, 66, 68–72, 74, 83, 84, 86, 87]. For instance, a woman in Australia disclosed her experience of being neglected after seeking care:

“*The midwife ring the department and told them that ‘oh the lady (…) the African lady is here, she been crying that oh she’s ready to give birth but water is not breaking. But now I have checked on her, the baby head is right there. She’s ready to give birth’. They [the medical staff] refuse … So they sent me home. When they send me home my baby pass away. Three days in my stomach … the day they [the medical staff] was ready for me to give birth the baby’s gone.*” ([54], p6-7).

#### Abuse

In this sub-theme, we noted experiences of verbal and physical abuse, as well as coercion by healthcare personnel around decisions about these women’s health. Moreover, we also included testimonies of racialized migrant women being put “on display*”* by healthcare personnel under this sub-theme.

Abuse was detected in 22 out of the 57 articles (22/57) [32, 40, 48, 49, 51, 53, 54, 59, 65, 67, 72–74, 76–78, 82, 84–88]. Abuse was identified in studies conducted in the US [51, 74, 85], Switzerland [65, 78, 88], Australia [43, 54, 77], Sweden [49, 88], Portugal [48, 88], the UK [67, 73], Canada [40, 76], Iran [53, 72], Türkiye [84, 86], Italy [88], Norway [88], Slovenia [88], Luxembourg [88], France [88], Germany [88], Croatia [88], Serbia [88], Israel [59], the Netherlands [32] and Jordan [82]. Among those studies, the majority used a qualitative methodology (18/22). The rest used quantitative (3/22) [40, 43, 88] and mixed-methods study designs [32].

An example of verbal abuse was reported by a migrant woman who gave birth in England: *“One midwife … she was rude to me, said, ‘Hey, why don’t you go back where you came from?’”* ([73], p4)*.* The above-mentioned abusive behavior is also captured in a quantitative population study from Australia by Yelland et. al [43], in which overseas-born women were more likely to report that “doctors and midwives talked down to them during labor and birth” (37.1%) and that “care providers were not always encouraging and reassuring” (49.4%) (667) in comparison with Australia-born women who were less likely to report abuse, with 25.3% and 34.5% respectively. Another quantitative cross-sectional study concerning the childbirth experiences of migrant and non-migrant women in Europe reported that 15% of migrant women suffered physical, verbal, and emotional abuse in comparison to 12.7% non-migrant women [88]. Moreover, healthcare personnel’s abusive behavior is captured in Rosanna’s experience as described in a study by Barata [48] within the Portuguese setting of obstetric care:

*“She had an unconsented episiotomy and ‘husband’s stitch.’ After stitching her through a painful procedure, because the effect of the epidural had waned and she could feel everything (but nobody paid attention to her complaints), the physician showed her husband that she had stitched a bit tighter.”* ([48], p6).

In addition, racialized migrant women reported that they felt coercion related to medical procedures and decisions regarding their health [32, 40, 48, 51, 65, 76, 78, 85]. A study [85] conducted in the US aiming to explore Somali couples’ perspectives on the care and support they received during the perinatal period included an indicative testimony of coercion and not being taken seriously, which was also apparent in the previous sub-theme:

*“At my last prenatal visit my doctor pressured me to get an induction. I said ‘I don’t feel my body is ready; it is my fourth kid, I would know,’ but I felt I had to agree. While getting the IV medicine, I was like, screaming: ‘something is terribly wrong, I have too much pain.’ It turned out my uterus broke (ruptured).”* ([85], p364).

Concerning being put on display, racialized migrant women in several studies [54, 67, 74, 76–78] mentioned that they felt used as study objects by students or that many health providers would visit them at the same time. In Australia, women with refugee backgrounds mentioned that healthcare personnel would walk in and out their rooms, while students would be brought to watch or practice procedures being done on them. One of them specifically shared: “*Other doctors and nurses would come in and that was really uncomfortable for me. They didn’t do anything, but they looked, and that was really uncomfortable*” ([77], p150).

#### Differential care

In this sub-theme. nineteen studies (19/57) [34, 35, 39–42, 44, 45, 55–57, 59, 72, 76, 77, 79–81, 88] were included. We noted the comparisons in treatment by healthcare personnel among various groups of racialized migrant women and between racialized migrant women and their local counterparts. The countries where differential care was identified were Norway [34, 35, 41, 88], Portugal [45, 88], France [79, 88], Germany [56, 88], Switzerland [80, 88], the UK [39, 55], Canada [40, 76], the US [44], Sweden [88], Italy [88], Slovenia [88], Luxembourg [88], Croatia [88], Serbia [88], Denmark [57], Israel [59], Iran [72], Australia [77], Austria [81], and Finland [42]. Six (6/19) [35, 39–42, 88] of those studies applied a quantitative methodology, and the rest used a qualitative one.

An indicative example of differential care was apparent in a quantitative study [35] examining factors associated with recently migrated women’s satisfaction with maternity care in Norway. It showed that refugee women (24.4%) felt treated differently by healthcare personnel because of their religion, skin color, and language compared to 9.3% and 13% of migrant women who migrated due to family reunification or those who migrated for work or education, respectively [35]. In Austria, a Japanese participant in Seidler’s et al. [81] study expressed her frustration when healthcare personnel treated her differently once they noticed that she could not speak German: “*I really had problems with those nurses who changed their attitudes once they knew I could not speak German.*” ([81], p8).

#### Consequences of discriminatory experiences

The last theme reflects the consequences felt and shared by racialized migrant women because of the experiences summarized in the two previous themes. Lack of agency and exclusion from decision-making are the two emerging sub-themes.

#### Lack of agency

In this sub-theme, e included limiting experiences (i.e., where one is unable to utilize or access maternal healthcare services) as an aftermath of discrimination. Lack of agency was evident in the majority of the included articles (42/57) [32, 35, 39–41, 43, 44, 47–49, 51, 53–57, 59, 62–78, 81–88]. The countries involved in these studies were Norway [35, 41, 63, 71, 83, 88], the UK [39, 55, 67, 68, 73], the US [44, 51, 70, 74, 85], Australia [43, 54, 77, 87], Sweden [49, 69, 88], Germany [56, 62, 88], Switzerland [65, 78, 88], Canada [40, 64, 76], Portugal [48, 88], Iran [53, 72], Denmark [57, 66], the Netherlands [32, 75], Türkiye [84, 86], Uganda [47], Italy [88], Slovenia [88], Luxembourg [88], France [88], Croatia [88], Serbia [88], Israel [59], Austria [81]and Jordan [82]. Thirty-five out of the 42 studies applied a qualitative methodology while six used a quantitative one [35, 39–41, 43, 88]. One study used a mixed-methods design [32].

In more than a third of the total number of articles (24/57)[35, 40, 41, 43, 47, 48, 54, 55, 63, 66, 68–72, 74–77, 84–88], racialized migrant women stated that they felt ignored or that their needs or expectations remained unmet. Additionally, in 15 studies (15/57) [32, 39, 43, 44, 54, 56, 64, 66, 67, 69, 72, 75, 76, 83, 85] participants disclosed that they avoided seeking care after having negative experiences or that they no longer trusted the services. Indicatively, a woman from Syria living in Denmark explained why she has no motivation to contact maternity services:

“*Three hours I am sitting freezing in the waiting room … no one comes and tells me I can lie down or brings me a blanket, nothing! I would rather avoid having to engage with them and ask them [maternity care providers] … it’s neglect of care, no one asks me if I would like to stay the night at the hospital … I am pregnant, I have no one, no network, someone beside me to take care of me.”* ([57], p10).

In a third of the studies (19/57) [44, 47–49, 51, 53, 56, 59, 65, 67, 69, 72–74, 78, 81, 82, 84, 85], racialized migrant women mentioned that they experienced feelings of shame and being stereotyped and judged or that they directly perceived discrimination when using maternity healthcare services. An Afghan woman living in Tehran recalled hearing a common refrain directed against Afghans: *“You are Afghanis; if your baby dies, you’ll come back next year with another*” [72]. Women reported feelings of helplessness and powerlessness in ten of the included articles (10/57) [32, 44, 47, 62, 65, 68, 73, 75, 77, 84]. An Arabic-speaking Syrian woman in Turkey expressed her helplessness when her birth started upon realizing that no one understood what she was going through: “*God, what am I going to do? They do not understand me, how will I get rid of this pain?*” ([88], p1176).

Seven articles (7/57) [44, 56, 67, 72, 82, 83, 85] included testimonies of fear, maltreatment, rejection, subjection, or having already been subjected to ridicule. Lina, a Syrian woman living in Jordan, was subjected to ridicule from the providers while seeking healthcare to give birth: “*Look at you, why do you want yet another baby?*” ([82], p6). In four studies (4/57) [49, 51, 53, 57], there was evidence that racialized migrant women had a fear of deportation because they sought care without being a legally documented entity in the host country: “*I didn’t go to hospital during my first pregnancy because my and my husband’s visas were expired and I feared if I go to hospital they will arrest us and send us back to Afghanistan*” ([53], p7). This could explain why in one article [73], there was specific mention that racialized migrant women’s overall well-being was negatively affected due to their lack of legal documentation.

#### Exclusion from decision-making

Exclusion from decision-making was noted in nearly one-third of the total collated articles (20/57) [33, 35, 38, 40, 43, 45, 48, 51, 54, 62, 65, 67, 68, 71, 72, 74–76, 85, 86]. This theme includes reports of absence of inclusion in decision-making processes, as well as lack of informed consent and choice regarding treatment or management. Portugal [38, 45, 48], the US [51, 74, 85], the UK [33, 67, 68], Australia [54, 87], Norway [35, 71], Canada [40, 76], Germany [62], Switzerland [65], Iran [72], the Netherlands [75], and Türkiye [86] were the areas included in these studies. Concerning the methodology applied in these studies, one was a multi-case study(33), four were quantitative studies [35, 38, 40, 87], and the rest were qualitative.

In one of Almeida et al.’s [46] studies conducted in Portugal, lack of informed consent and non-involvement in decision-making was clearly illustrated in an African participant’s experience with a doctor attempting to impose a subcutaneous implant on her: “*How can I put one thing if I don’t know what it was? She didn’t even ask me for my opinion; she didn’t ask me if I wanted it! The answer she gave me was ‘Oh, it’s for you not to become pregnant again.*’” ([46], p336).

## Discussion

This scoping review aimed to look at the various discriminatory experiences of racialized migrant women when accessing maternal healthcare by gathering evidence from empirical literature. The included articles, most of which were qualitative studies conducted in mainly European and North American countries, expose some of the ways that racialized migrant women experience discrimination while utilizing maternal healthcare services in their host countries. From being rendered invisible, experiencing suboptimal healthcare services, and perceiving that they were being stereotyped because of their migrant status, these varied experiences were united by the common theme of discrimination. Those perpetrating these disrespectful and discriminatory acts, whether unconsciously or not, were the healthcare personnel the women were directly interacting with during their perinatal care. While the included articles allude to some of the issues related to discrimination in maternal care by racialized migrant women, our scoping review delineated knowledge gaps that warrant discussion. These knowledge gaps are as follows:

- The limited geography of research and knowledge creation on discrimination
- The lack of articles focusing on and conceptualizing discrimination and racialization in maternal healthcare
- The lack of an intersectional lens in exploring discrimination against racialized migrant women in maternal care

### The limited geography of research and knowledge creation on discrimination

The majority of the available studies concerned Western countries, also referred to as the Global North. According to Czaika and Reinprecht [89], there are various migration drivers—including individual, group, macro-structural, and external—that are behind the reasons why people decide to migrate. One of these is based on the individual’s finances and their country of origin’s economy. The wage differences and higher income levels, usually in European and North American countries, attract migrants from countries considered to be part of the Global South. Nevertheless, at the same time, it is not the poorest of those countries who can actually migrate—reflecting an inverse U-shape between migration, development, and poverty [90]. On a more macro-level, migration is also driven by the current global capitalist system whose structure is closely linked to colonial histories and structures. Czaika and Reinprecht [89] explain in their work that the global demand from more corporate capitalist (predominantly Western) powers for a steady supply of cheap and flexible labor—usually from less-resourced countries that have been historically part of former colonized parts of the world—drives domestic and international migration patterns that eventually destroy local economies and traditional livelihoods. Outside of economic discussions and seemingly more voluntary movements, migration is also influenced by conflict and political interests. For those seeking asylum or refuge away from where they are persecuted or experience suffering due to local authoritarian powers, where they can migrate to is also still controlled by political powers but on a more global scale. When the European Union created the European Asylum and Immigration Pact, it established universal policies that allowed European powers to control and limit migration through eroding the humanitarian aspect of asylum, tightening migration restrictions; and shifting to an economic demand for labor migration, therefore dictating where migrants are allowed to stay [91].

Regardless of the driving factor, this more popular move to countries with higher income levels created a need to conduct this type of migrant health research to ameliorate the existing host country’s healthcare according to the needs of non-native populations and based on their public policies of providing existing social benefits to all living in their country. However, the lack of similar research in non-European or non-North American (i.e., Global South) countries that, likewise, experience high migration rates also highlights the asymmetry in research—described as epistemic injustice—between high-income countries and low- to lower-middle-income ones [92]. Besson [92] has stressed that fewer funds are provided to non-Euro-North-American countries and institutions, resulting in the dominance of Westernized knowledge and the continuation of the colonial mindset in global health [93].

### The lack of articles focusing on and conceptualizing discrimination and racialization in maternal healthcare

A conclusion from our review regards the scarcity of research on discrimination against racialized migrant women, which would allow us to draw from evidence and provide solutions to undoing the discrimination they face in maternal healthcare. This is mainly because the included research is fragmented and does not focus on exploring discrimination in maternal healthcare. Among the 57 included studies in this scoping review, none of them initially aimed to conduct a discrimination study. All articles mainly focused on researching the varied experiences and perceptions of racialized migrant women in maternal healthcare. Nevertheless, racialized migrant women described their experiences of discrimination when accessing maternal care services, which draws attention to the importance of exploring these experiences further.

That the included articles did not solely focus on exploring discrimination in maternal healthcare by racialized migrant women could be partly attributed to the fact that healthcare research has been historically formed by discriminatory ideologies [94], such as colonialism, racism, classism, and sexism. Thus, addressing and challenging the existing system within it seems demanding and daunting for those working in this specific area. Moreover, public health and the field of medicine continue to highlight (mainly Western) healthcare and their personnel as highly rational and neutral such that interactions, diagnosis, and treatments are perceived as based on these notions rather than on any potential bias [95]. This perception continues to be upheld, even with the existence of research that points to the existence of bias against racialized groups that influence various dimensions of care [4]. Moreover, the fact that discrimination against racialized groups is often implicit and driven by unconscious biases and stereotyping makes it difficult to assess and monitor the actual experiences and impact of discrimination in healthcare settings [96].

Furthermore, the lack of focus on discrimination against racialized migrant women could also be attributed to a lack of conceptualizing discrimination and discriminatory practices. Racialized migrant women in the included studies expressed feeling that their inputs from previous birthing experiences were being disregarded or that they were being put on display because of their physical differences (for instance because of Female Genital Circumcision) from their local counterparts—these reflect how racialized minority identities and practices become risk factors to experiencing mistreatment while accessing maternal healthcare [97]. Feelings of being ignored, receiving poorer quality healthcare services, and experiencing being stereotyped a certain way are all products of a system that perpetuates a bias—whether subconscious or not—of who matters and deserves respectful treatment [93]. However, the experiences of these women were not put into a historical context of racialization. In obstetrics and gynecology, racism has played a fundamental role in creating certain practices and knowledge [98]. Racism, according to Shannon, et al. [93], has also been instilled in healthcare personnel during their professional training and education through perpetuating whiteness and maleness as the norm. Most of the articles are from Western settings, where migration and the category of the migrant are often racialized as non-white and non-Western [99] which entails viewing migrants as problematic and non-compliant healthcare users compared to their White counterparts [99, 100]. The racialization of the migrant has become, particularly recently, a hegemonic public and political discourse, which constructs migrants as lazy and exploitative of the welfare system [101]. In healthcare, the racialization of racialized migrant groups is seen for instance in European contexts where racialized users are constructed as bad users and their health complaints as unworthy of care, subsequently resulting in suboptimal and differential care [100]. This racialization is left unexplored in the included article even though studies on racism and racialization in healthcare report similar experiences by other racialized groups, such as Black and Indigenous populations. Certainly, the fact that perinatal and maternal mortality, as well as other maternal health outcomes such as stillbirth, are reported to be higher among racialized migrants and even higher among women Black women and other racialized women alludes to racializing tendencies [102], requiring further investigation into racialization and racism in maternal care.

The idea that healthcare is supposedly neutral and objective also acts as a barrier to investigating healthcare personnel’s racial bias against racialized migrant women, evidence of its existence and association with various diagnoses and treatment choices notwithstanding [4].

To better understand why discrimination and racialization processes of care in maternal care persist, it is important to not just document racialized migrant women’s experiences; rather, it is essential to examine healthcare personnel’s racial biases and attitudes. Our findings have shown that healthcare personnel, particularly physicians and midwives, at the highest level of influence and authority in the health service setting demonstrate racialized biases towards racialized migrant women. As these healthcare personnel constitute the main providers responsible for racialized women’s well-being, and given the significantly high mortality and morbidity rates racialized women face in maternal care [102], examining providers’ biases is essential. It is also crucial to examine existing power relations and racializing care processes that may exist in healthcare settings. As physicians are often located high in medical hierarchies, ignoring these aspects of racialization may also have an effect on anti-discriminatory/racist interventions, which may be hindered through methods of denial rooted in healthcare neutrality [103].

### The lack of an intersectional lens in exploring discrimination against racialized migrant women in maternal care

The patriarchal culture existing within obstetrics and maternal care results in obstetric violence, which derives from the notion that women’s bodies are of a lower status [104]. Our results demonstrating the abuse and mistreatment [32–35, 40–42, 47, 49, 58, 63, 65, 67, 69, 71–74, 79, 80, 83, 87], as well as the overall disrespectful practices, experienced by migrant women are consistent with other studies that prove the systematic nature of obstetric violence [97, 105, 106]. Our results also allude to the racialization of racialized migrant women. The included studies in this scoping review showed that discriminative encounters are an unfortunate part of the maternity care experience of migrant women. Moreover, what cannot be ignored is the fact that discrimination is not attributable to just one factor. On the contrary, it is based on how the different identities that migrant women have intersect with one another and are perceived within the maternal healthcare system. The combination of discriminations is captured by the intersectionality theory articulated by Kimberlé Crenshaw [107]. According to the theory, multiple aspects of a person’s identity and existence intersect and create different modes of discrimination or privilege. When summing up these identities on a structural level, the result is that racialized migrant women are placed in the least powerful position within a highly hierarchical system. Applying an intersectionality lens—that is, understanding that the person is female and from a racialized group and a lower economic sector—creates a distinct point of intersection, helping to understand how racialized migrant women face discrimination and violence [108]. Taking into consideration the amount of evidence in our study that is consistent with other systematic reviews [109–111], institutions seem to perceive racialized migrant women as incapable of having ownership over themselves such that they are perceived as unable to make decisions about their bodies and are viewed as unentitled to their right to informed consent or privacy. Therefore, under the guise of providing care, systems and institutions paternalize and oppress women becoming systems of oppression rather than systems of care. As part of the maternal care experiences of racialized migrant women, not only structural discrimination—like racism and xenophobia—arise but also communication barriers arise [111], thus adding further challenges to their healthcare using process. Another axis of inequality stems from one’s socioeconomic status. Lower socioeconomic status is interrelated with lower life expectancy and increased rates of mortality and morbidity [24]. This intersectional nature is highlighted in our review through the evidence demonstrating that racialized migrant women were highly discriminated against because of the maternal healthcare system’s biased perceptions of their racialized identities, migrant status, sociocultural position, and biological sex together with the hierarchical way it operates. However, there is a lack of an intersectional analysis in the included articles that would situate analysis of the added disadvantages that coexist with being a racialized migrant woman, who is already part of a discriminated population yet still faces further facets of discrimination while accessing maternal healthcare in a supposedly safe environment.

### Methodological limitation

The results of our review must be considered alongside the limitations brought by our chosen methodology. As it is a scoping review, the number of included materials was large and methodologically diverse. Our time constraints also limited our ability to include gray and literature not published in scientific journals. Thus, the interpretation of the comprehensiveness and quality of the studies here should be made with caution [28].

Our inclusion and exclusion criteria may have also significantly affected the outcome of our research. Having a restriction for the timeframe of the published articles, only including English peer reviewed articles, and omitting other reviews and gray literature may have resulted in our study missing additional insights from older, relevant and non-academic literature. Further, overlooking important diverse experiences that have been published or written in other languages is also a limitation that may have emphasized the already existing hegemony of the English language rooted in historical imperialism. Excluding literature with more specific populations under the umbrella term “migrant women”—such as internally displaced or dispersed migrant women; those living in refugee camps or detention centers; and adolescent migrant mothers—may have also omitted vital findings that could have shed a different light on more intense experiences of discrimination. We recognize that these additional identities may subject migrant women to even more extreme discriminatory conditions that disrupt their utilization of maternal healthcare services [112–114].

The thematic categorizations made in this research must also be deliberately interpreted, as these were done according to the inspiration we got from the designs of thematic analyses [30] and what would be easiest for the readers to understand—rather than using an established framework. Thus, readers should be aware that the themes inductively developed on discrimination were based on our subjective interpretations of the content of the included studies.

## Conclusion

While our scoping review alluded to evidence highlighting experiences of discrimination by racialized migrant women in maternal care, this evidence is restricted to Western contexts. The Western dominance in research does not only reflect the migration movements but also the concentration of funds in the West contributing to the hegemony of research in these settings. The review also highlights the lack of evidence in research focusing on and conceptualizing discrimination and racialization in maternal care including research examining healthcare personnel’s racial biases and beliefs. Moreover, the lack of conceptualizing discrimination and racialization is coupled with a lack of an intersectional perspective emphasizing racialized migrant women’s experiences in maternal care.

It is of high importance that research addresses and explores the gaps highlighted in our scoping review; otherwise, healthcare risks producing discriminatory and racialized processes that are detrimental to racialized women’s livelihoods and health. To achieve this, we recommend that future research:

- Addresses discrimination and racialization directly and comprehensively;
- Stems from the fair distribution of research funds;
- Is multidisciplinary and focuses on deconstructing the systemic oppression in healthcare systems; and
- Accommodates intersectional approaches when researching discrimination and racialization in maternal care.

## Supplementary Information

Supplementary Material 1.

## Data Availability

No datasets were generated or analysed during the current study.
